# An engineering perspective of vacuum assisted delivery devices in obstetrics: A review

**DOI:** 10.1177/0954411920956467

**Published:** 2020-09-15

**Authors:** Dushyant Goordyal, John Anderson, Ali Alazmani, Peter Culmer

**Affiliations:** 1 University of Leeds Faculty of Engineering, Mechanical Engineering, Leeds, West Yorkshire, UK; 2 Bradford Teaching Hospitals NHS Foundation Trust, Bradford, West Yorkshire, UK

**Keywords:** Vacuum assistive delivery, birth, obstetrics, neonatal trauma, maternal trauma, ventouse, vacuum extraction, operative vaginal delivery

## Abstract

Complications during childbirth result in the need for clinicians to use ‘assisted delivery’ in over 12% of cases (UK). After more than 50 years in clinical practice, vacuum assisted delivery (VAD) devices remain a mainstay in physically assisting child delivery; sometimes preferred over forceps due to their ease of use and reduced maternal morbidity. Despite their popularity and enduring track-record, VAD devices have shown little evidence of innovation or design change since their inception. In addition, evidence on the safety and functionality of VAD devices remains limited but does present opportunities for improvements to reduce adverse clinical outcomes. Consequently in this review we examine the literature and patent landscape surrounding VAD biomechanics, design evolution and performance from an engineering perspective, aiming to collate the limited but valuable information from a disparate field and provide a series of recommendations to inform future research into improved, safer, VAD systems.

## Introduction

**Figure 1.**
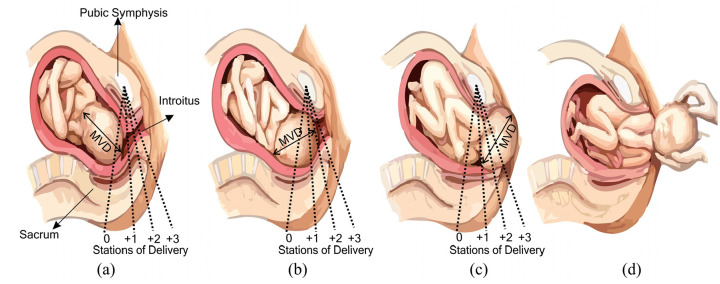
Timeline of normal delivery events: (a) Foetus aligns to the bony maternal pelvis in a cephalic presentation(vertex/head first), (b) Baby progressing through the stations of delivery, (c) Baby’s head scalp is visible at the introitus, and (d) Delivery of the baby is completed where the body delivers, either spontaneously or with the healthcare professional (accoucheur) holding the foetal head, sometimes to help delivery of the shoulders. This also marks completion of the second stage of labour.^4^

Since 1990, there has been significant improvements in maternal and foetal outcomes during childbirth.^1^ Across the world, most childbirth occurs naturally, that is, without physical assistance. Non-assisted cephalic delivery, in which the baby’s head emerges first, accounts for nearly 95% of all births.^2,3^ In this situation, the mother’s expulsive efforts, combined with the contractive force of the uterus, provide a coordinated motive force to push the baby from the uterus, along the birth canal. This is described clinically in terms of the baby’s descent through the pelvis, marked by ‘station’, as shown in (Figure 1), until the head initially ‘crowns’ (the foetal scalp at the vertex becomes visible between the labia minora, at the introitus) and then delivers on the perineum.

Unsurprisingly, the clinical and biomechanical aspects of childbirth have been extensively studied, see for example,^5,6^ and it is evident that despite being an everyday occurrence, natural childbirth remains a challenging event for mother and child alike. As a consequence, complications can occur which impede normal vaginal delivery, or require the delivery to be expedited with common factors including narrowing of the birth passage, foetal distress, intrapartum infection, maternal exhaustion or abnormal positioning or size of the baby.^7,8^

If normal spontaneous vaginal delivery is not possible, or needs to be expedited and the labour has entered the second stage (the cervix is now fully dilated to 10 cm), the obstetric team have two main options; providing additional assistance to the mother through Instrumental Vaginal Delivery, or performing a second stage caesarean section (C-Section).^9,10^ However, the C-Section is typically only used as a last resort (when if instrumental delivery is not safe and achievable or fails) because it can risks significantly greater maternal morbidity (more blood loss and postnatal aftercare).^11,12^ In the UK, all caesareans which are not planned electively, are classified as an emergency, though the RCOG.^13^ Clinical guidelines provided by National Institute for Health and Care Excellence (NICE) indicate on advisory classifications of how urgently each emergency needs to be treated.^14^ By this definition all caesarean sections done in the second stage, that is, after full dilatation of the cervix, are emergencies. This definition may not be applicable to other settings outside the UK. Even in the absence of foetal distress, a multitude of other emergent maternal indications for caesarean section exist, for example severe pre-eclampsia, severely growth restricted babies, or significant antepartum haemorrhage. Even excluding these emergencies, prolonging the second stage of labour can bring additional maternal morbidity (subsequent pelvic floor dysfunction and lifelong associated health problems and increased rates of post-partum haemorrhage) and therefore such second stage procedures cannot be truly regarded as purely elective.

Instrumental Vaginal Delivery is a clinical means of providing additional mechanical assistance to the mother’s contractions, providing both additional force and guidance to baby during the second stage of labour (when the mother’s cervix is fully dilated to 10 cm). Instrumental delivery comprises two families of techniques: Obstetric Forceps delivery and Vacuum Assisted Delivery (VAD) and is performed by trained obstetric professionals (accoucheurs). Combined these have a reported usage in 12% to 15% of registered deliveries in the UK^10,15^ and approximately 5% in USA.^16^ Both procedures help to achieve spontaneous delivery by providing augmented force to uterine contractions and maternal expulsive efforts and in cases of malposition, to correct the position of the foetal head, enabling passage of the foetal head through the pelvis. Specialised obstetric forceps can actively rotate the foetal head and subsequently aide delivery. Forceps can also be used after the manual rotation of the foetal head by the accoucheur. VAD also aids proper spontaneous rotation of the foetal head. It is worth noting that the correct application and use of the forceps should not require significantly more traction force as compared to VAD, nor should this be applied if the foetal head is misaligned. While it is possible to transmit more force to the foetal head using the forceps compared to VAD this should only have application in the event of acute time-critical emergencies where delivery is mandated, or when maternal exhaustion compromises the quality of expulsive maternal effort and makes the VAD more likely to fail.

Accoucheurs are trained as per professional body guidelines such as the USA & UK College of obstetricians & gynaecologists (ACOG or RCOG) to identify the prerequisites for instrumental vaginal delivery.^17^ In addition, simulation-based techniques such as mannequins or computational visualisation are used to complement or improve their proficiency.^18,19^ Instrument selection is driven by the clinical training received to identify and deal with complicated birth scenarios and some of the factors relating to choice of instrument outlined above.^20^ Other contributing factors are linked to the stations of delivery and orientation of the foetal head (foetal occiput anterior, or posterior or transverse). VAD can be preferred by proficient accoucheurs proficient in their use, over the forceps for low cavity (+3) as well as occiput posterior mid-cavity (0 to +2) delivery but there is no clear preference for normal mid-cavity delivery.^21^ The station of the presenting part of the baby (in the case of an instrumental assisted delivery, as a prerequisite, this will be the foetal head i.e. a cephalic presentation) is usually determined by digital vaginal examination by a trained birth attendant, who palpates the leading edge of the presenting part in relation to a bony anatomical landmark in the maternal pelvis, the ischial spines. These mark the mid position (or plane) of the anatomical ‘true’ pelvis and can subsequently be used to mark the progress of the decent of the presenting part through the pelvis. This is conventionally measured in centimetres, above (minus) or below (plus) the ischial spines. The clinical description ‘–3 above spines’ would therefore represent a high head, the leading edge of which is only just entering the maternal true pelvis. ‘+1’ represents a head which has advanced 1 cm beyond the plane of the ischial spines. There are three classifications for acceptable and safe operative vaginal deliveries (mid, low and outlet), which can be described in terms of the station below the ischial spines. Commonly three and sometimes five stations are used to describe advancement of the presenting part below the spines. As such a mid-pelvic delivery is 0 to +2 in station; low pelvic delivery is more than +2 but above the maternal pelvic floor. An outlet instrumental delivery occurs when the head is crowning or the foetal scalp can be visualised without separating the labia^17,22^

**Figure 2.**
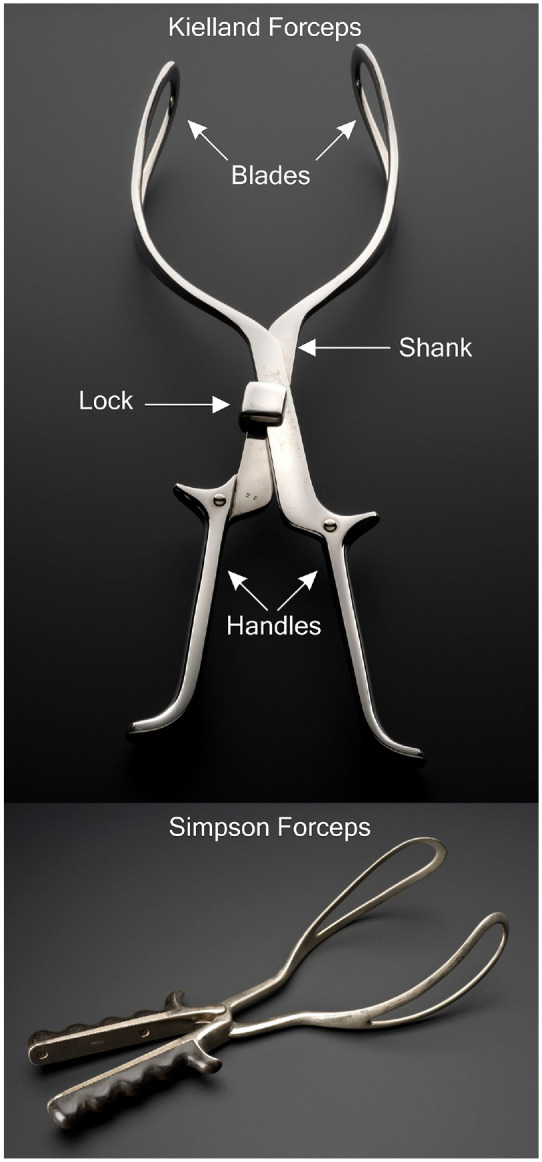
Illustration of Forceps. Top: Key design features of Kielland Forceps,^36^ Bottom: Illustration of Simpson Forceps.^37^

Modern obstetrical forceps, usually made of stainless steel, were first introduced in the 16th century to help assist troublesome childbirth. Key design elements feature a curved blade, shaped to match the contours of the baby head and maternal pelvis, provide easy manoeuvrability through the birth canal. Forceps are available in a wide variety of designs to accommodate differing delivery needs, as shown in Figure 2. For example, Simpson’s forceps are widely used for outlet deliveries because they conform well to the baby’s head, Keilland’s forceps are used to assist rotational delivery due to their narrow profile. Closed blade systems like Simpson-Luikart forceps were designed to conform to the curve of the maternal pelvis (cephalic pelvic curve).^23–26^ The use of the forceps requires extensive training but and remains clinically challenging,^27^ with links to increased maternal morbidity (e.g. anal sphincter injury) and cosmetic damage to the baby’s head.^28^ Case reports and litigation relating to alleged improper use of the forceps report rare but catastrophic severe bony injuries (skull fractures) and poor foetal outcome. As a possible consequence of this, combined with preferences in training of obstetricians, the use of VAD devices has increased in the past decade, viewed by some as a less traumatic alternative to forceps.^29^ This is not necessarily true, as use of VAD has higher risk of cephalohematoma and subgaleal haemorrhage than forceps. Improper use (incorrect positioning, incorrect direction of traction and multiple re-applications of the cup) increase this risk further. However, it is important to note that although rare, cephalohematoma, subgaleal and even intracranial haemorrhage can also occur in spontaneous vaginal delivery. While each type of instrument has advocates, general obstetric opinion does not favour one type over the other. Instead, training advocates use of the correct instrument in the appropriate situation, balancing the possible maternal and foetal risks against the need to deliver and the precise indication in each circumstance. Such decision making is at the core of the skills of every good obstetrician. The need for this is clearly outlined when scrutinising the use of sequential instruments (VAD devices then forceps), which is clearly associated with higher rates of foetal morbidity. This occurs when the VAD is used and fails to deliver the baby and the forceps are subsequently used to complete the delivery. Owing to the differences in the traction which can be applied using the forceps, a delivery can then be completed where VAD has failed, however it carries higher risks. Another alternative would be to perform a caesarean section, however if the foetal head has been brought to a low or outlet station by instrument(s), freeing the deeply engaged foetal head can be extremely difficult, resulting in additional trauma to the uterus, significant bleeding and in extreme cases, hysterectomy and admission to intensive care.^30^ Stellate foetal skull fractures are also reported as a consequence of full dilatation caesarean section where the operator and an assistant have attempted to dis-impact the foetal head (the latter pushing upwards from the vagina).^31–35^

Use of each instrument is therefore part of a balanced judgement made by obstetricians on a daily basis, weighted around foetal position, size, progress in labour, quality of maternal effort, foetal head position, personal proficiency and skill and the perceived likelihood of successful delivery.

VAD was brought into widespread clinical use through the introduction of a system proposed by Malmstroem,^38^ aiming to impart assistive forces through a suction interface on the baby’s head. In general the VAD consists of a suction cup which is placed on the scalp, a negative pressure is then applied (either via manual or electric pump) such that the accoucheur assist by pulling the VAD handle in tandem with the mother’s contractions.^39^

Since the original VAD device from Malmstroem, there has been little evidence of innovation in device design or function. While this is not uncommon in surgical instrumentation it should be considered in the context of growing clinical evidence that VAD systems could, and should, be improved for improved safety and efficiency. Unfortunately, our work has found that the research base of technical literature and information regarding VAD systems is scarce, disparate and often difficult to source, ultimately acting as a barrier to innovation. Therefore, our aim in this paper is to collate and review key clinical and technical literature on VAD systems, placing a particular emphasis on providing an engineering perspective to inform and inspire future improvements in the field. We begin the review by considering clinical use and requirements associated with VAD devices, using this as a foundation to discuss their design evolution, before exploring research into VAD mechanics and performance. We conclude with a discussion which highlights gaps in the knowledge base and key opportunities for future innovation.

## Clinical use of VAD systems

Clinical indications for instrumental delivery include (and are not limited to), a prolonged second stage of labour, maternal exhaustion, foetal intolerance of labour (‘foetal distress’ signified by abnormal cardiotocography (CTG), delivery of a second twin, pre-eclampsia or eclampsia, intrapartum infection or significant antepartum haemorrhage. If a birth requires an assisted delivery, the clinician must review several prerequisites before they proceed. The prerequisites of any assisted vaginal delivery are that the cervix is fully dilated, foetal head position and station has been determined (and that the station is below the ischial spines), adequate analgesia, emptying the maternal bladder, patient consent, a willingness to abandon the procedure, and an alternative method of delivery (caesarean section) if the assisted vaginal delivery fails. Preparation for this final consideration may include moving from the delivery room (where some instrumental deliveries can be performed e.g. very low (+3) direct occiput anterior ‘lift-outs’) to the operating theatre where an instrumental delivery can be attempted, with preparation being made to effect a prompt caesarean section in the event the attempt at instrumental delivery fails. If there are significant concerns (e.g. regarding estimated foetal weight, significant malposition, high station, or risk factors for severe should dystocia) such that the delivery is likely to fail, or the opportunity to cause significant harm is deemed high, then it may be necessary to reconsider the planned mode of delivery as shown in Figure 1.^17,40^

**Figure 3.**
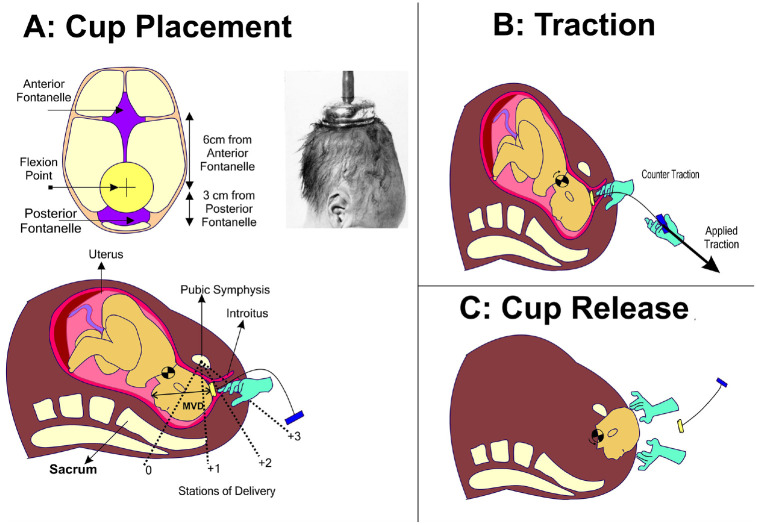
Process steps during VAD: (a) Cup placement-A vacuum source is applied to create a chignon by manual/electric pumping after placement on the flexion point. On caption-Illustration of Malmstroem's cup placement on a foetal head,^38^ (b) Traction-Applied traction with a counter traction used to overcome resistant introitus and (c) Cup Release: VAD device is released to proceed with delivery of the head.

After meeting those prerequisites, the VAD device can be applied onto the baby’s head. The first step is critical in which the clinician must identify the correct location for VAD attachment on the baby’s scalp; the flexion point is located 3 cm anterior to the posterior fontanelle along the midline of the sagittal suture, as shown in (Figure 3).^5^ The VAD device is then manoeuvred through the delivery channel and onto this point and a vacuum is applied to create a secure attachment with the scalp. This differential pressure with the atmosphere causes the first intermittent layers of the scalp to expand outwards from the aponeurotic galea to fill inside the cup. The result is an elevated region of scalp filled with fluid, known as the caput succedaneum chignon, a type of localised oedema (or colloquially as a ‘chignon’ or ‘localised oedema’) which forms a mechanical scalp-device interface,^7,41–43^ see Figure 5. Generally, oedema is the result of any serous fluid collection in tissue and can be the result of multiple causes for example, infection, inflammation or trauma. During labour, the serosanguinous fluid accumulating in the subcutaneous tissue of the foetal scalp and the periosteal tissue of the foetal skull, is like oedema but termed caput succedaneum. Though in practical terms, caput and chignon are the same, it is important to differentiate the chignon, which is a collection of serosanguinous fluids induced by the vacuum, from the caput succedaneum, which is a natural collection of fluid (sometimes serosanguinous) induced by labour where the foetal head presses against the dilating cervix.

After the chignon is formed and held, the VAD device can be employed by the clinician to assist the mother using the VAD handle during each maternal contraction. This process has one main aim; to assist descent (movement) through the birth canal. If the foetal head is malpositioned away from the ideal occiput anterior position, the traction exerted through the correctly positioned VAD causes flexion of the head and descent through the maternal pelvis, promoting spontaneous rotation of the malpositioned foetal head to the occiput anterior position. The clinician angles each pull to promote flexion of the baby’s head, bringing the chin towards the chest and orientating the occipital end of the scalp towards the pelvic outlet^44^ (Figure 2). Full flexion is achieved when the ‘Mento-Vertical Diameter (MVD, the vector between the chin and VAD flexion point) points towards the entrance to the birth canal.^44,45^ The procedure typically lasts around 10 minutes over 2 to 3 pulls, each exerting a force up to 115N. This process achieves a success rate of over 80% when used with a commonly available VAD device (Kiwi OmniCup™, shown in Figure 7).^46^

**Figure 4.**
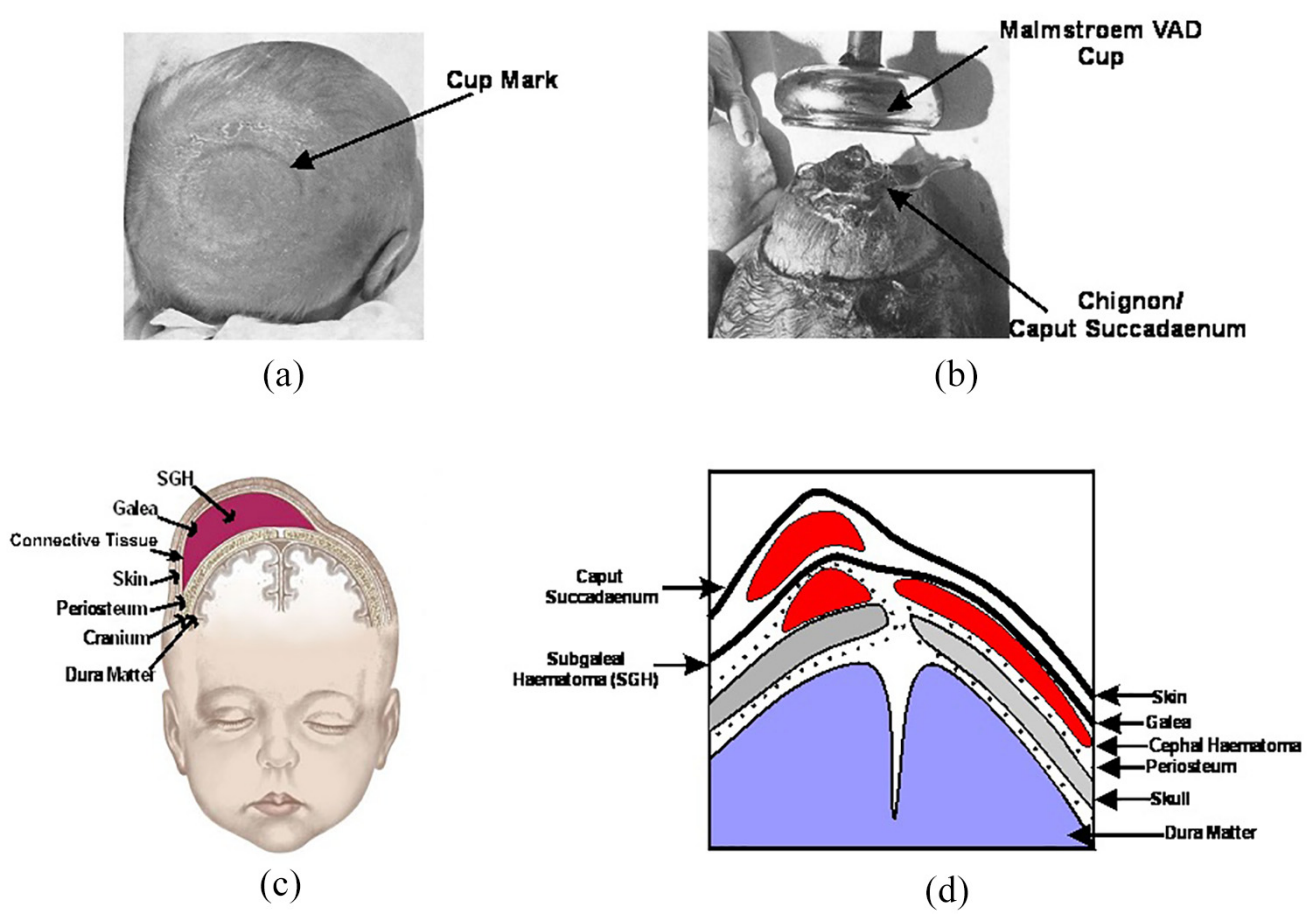
Trauma associated with VAD: (a) Elevation of scalp after VAD,^38^ (b) Dissipation of caput succedaneum after a few hours leading to a cup mark^38^ (c) Baby head with SGH^47^ and (d) All trauma levels associated with VAD.

The position of the baby is constantly monitored by the clinician’s other hand. In some cases, a ‘counter-traction’ is applied, a force opposing the main direction of movement in order to maintain device position and orientation during traction. Bird demonstrated that traction was a two-handed exercise: ‘The thumb of the non-pulling hand, pressed firmly against the cup near the rim, helps to prevent the cup from tilting off the scalp. The index finger of the non-pulling hand, resting on the shoulders of the cup with its tip touching the scalp, monitors descent.’ The index finger was used to detect descent of the scalp without descent of the bony skull – negative traction as Bird called it, and a sign of unyielding obstruction.^45^ This technique is also reported to help the clinician gauge and regulate the tractive force, particularly during outlet deliveries when the foetal head must pass through a narrow (and thus restrictive) introitus^46,50^ (Figure 4). The VAD device is used until the appearance of the baby’s head past the introitus, termed ‘crowning’. At this point further assistance is typically not required since the baby’s head represents the most significant resistance to movement during the birth process. Ending use of VAD consists of releasing the vacuum after the sight of delivery of the baby’s chin. The VAD can be used until the delivery of the foetal head, as the foetal head usually represents the most significant resistance to the force generated by uterine contractions and the expulsive efforts of the woman. The VAD, if used to assist delivery, is removed after the delivery of the foetal head, signified by the emergence of the foetal chin from the introitus.

In some cases, a VAD is required to assist in rotational delivery which involves addressing a malposition of the baby’s head. This follows the same basic procedure described above but with a redirection of traction according to the station of delivery and always along the axis of the maternal pelvis.^39^

**Figure 5.**
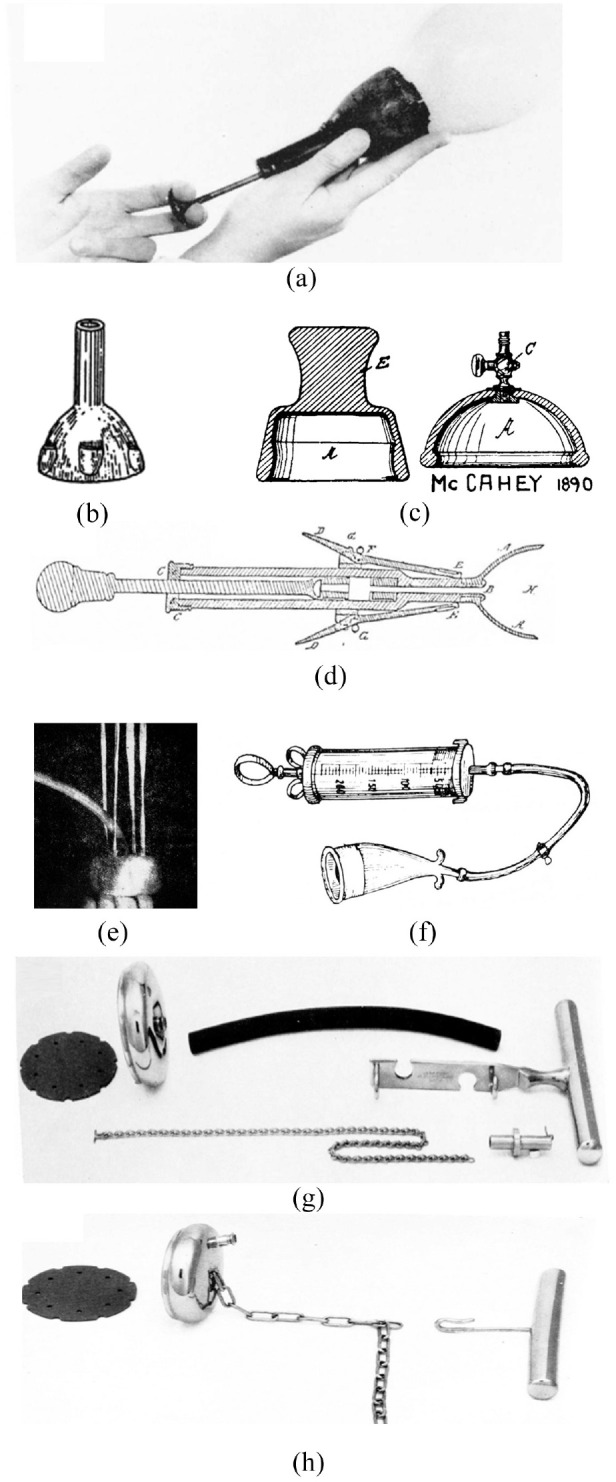
The evolutionary track of VAD device design: (a) James Young Simpson’s ‘Air Tractor’,^48^ (b) Saleh’s rubber cup with finger grips,^49^ (c) McCahey’s designs, (d) Stillman’s design,^49^ (e) Couzigou’s ventouse eutocique,^48^ (f) Finderle’s horn VAD device,^48^ (g) Malmstroem’s VAD device proposed in 1968,^48^ and (h) Bird’s modified VAD device proposed in 1969.^48^

Despite being an established instrument in labour wards across the world, there remain safety concerns behind the use of VAD devices. The chignon created by the vacuum action of the VAD device, shown in Figure 5, creates a striking visual impression of trauma, but in actuality it typically only persists for a few hours to a day, before dissipating, with associated cup-marks healing over a period of days. The adverse events which cause more profound trauma to the baby are less common, but also less visually apparent, making detection challenging. The mechanical interaction between VAD device and scalp can result in damage to the underlying scalp anatomy to varying degrees.^51,52^ Subgaleal haematomas (SGH) occur in approximately 6 in 10,000 VAD deliveries, when excess blood from the emissary veins accumulates beneath the epicranial aponeurosis (galea). This requires immediate attention as the blood can spread across the entire calvarial vault. If not diagnosed promptly, the resultant blood loss could lead to a life threatening hypovolemic shock (a 1 cm depth increase in subgaleal space could accommodate up to 260 mL of blood,^53–55^ approaching the circulation volume of a 3 kg baby.^56–58^ The occurrence of SGH is strongly linked to inappropriate cup placement in VAD.^17,59^ In the majority of SGH cases, incorrect cup placement such as the leading edges of the cup were located too close to the anterior fontanelle (less than the recommended 30 mm)^60^ and even small errors in placement can lead to severe injury.^61^ Another notable adverse event in VAD use is unintentional cup detachment (often termed ‘pop-offs’).^62,63^ This is problematic firstly because it can cause, or exacerbate, head trauma to the baby (as noted in Scalp abrasions, Cephalohaematomas and SGH) but also because it can impose a profound change in the delivery plan; in the UK, after two to three pop-offs have occurred the delivery team must abandon the delivery to opt for a second instrument or revert to a caesarean section with significantly higher risks of morbidity and poorer outcomes for mother and baby alike. During the past decades, there have been a significant effort to mitigate the clinical risks through the use of different materials, design and instrumentation as detailed in the next section.

## Design evolution of VAD devices

Given the wide use of VAD systems for assisted delivery it is instructive to consider how VAD device design, function and performance has evolved since their inception.

Although wide-scale use of VAD techniques has only occurred since the 1950’s, the concept and early prototype systems have been in existence far longer. In 1848 Sir James Young Simpson, inventor of the Simpson’s forceps (Figure 2) also proposed their alternative; the ‘Air Tractor’ can be credited as the first VAD device, motivated in an effort to reduce maternal trauma (Figure 5).^64^ The device comprised of a brass syringe attached to a 3-inch diameter cup made of vulcanised rubber covering a metal insert. Entry to the cup was covered by a brass wire gauze where a piece of sponge or flannel was housed to inherently prevent obstruction of the vacuum inlet.^65^ The device wasn’t widely adopted due to reported concerns behind its limited suction force leading to Simpson to concentrate on the commercialisation of his forceps invention. Despite not being popular in the UK, Simpson’s work inspired others. In 1886 French inventor Soubhy Saleh produced a rubber cup connected to a separate vacuum pump while in the USA Stillman patented a VAD-like device in 1875 comprising of an oval cup with collapsible rings to facilitate entry, coupled to a traction handle.^49^ The ‘Atmospheric Tractor’ from McCahey followed in 1890 featuring a near-hemispherical rubber cup which was depressed, much like a plunger, onto the baby’s head without an external vacuum pump.^38^ In 1912 Kuntzsch developed the ‘vakuumhelm’ which employed a manometer to gauge the vacuum level inside an attachment cup. This was used in two successful trials on still-born infants but, like the devices preceding it, was not developed or used clinically.^41^

It was only after several more decades and the introduction of the ‘ventouse eutocique’ device in 1947 that VAD devices achieved clinical recognition.^66^ This device consisted of a straight sided aluminium cup (diameter 40–65 mm) and a braided pull cord for improved angular manipulation.^67^ Vacuum was generated in the cup using an electric pump which included a waste trap for amniotic fluid and blood.^68^ A similar approach was patented by Finderle in 1952, albeit with a horn-shaped cup, but despite a reported 221 successful cases the device was discontinued.^69^ However, it was the introduction of Malmstroem’s VAD system in 1953 which brought more widespread clinical use and closely represents those systems used today.^70^ Malmstroem produced an improved design in 1957.^38^ The latter consisted of a vacuum cup with a curved cross-section (diameter 33–60 mm), designed to create a mechanical interlock with scalp tissue when a vacuum was applied through an external pump. Traction is controlled by metal chain and handle to the cup. While it represented a step-change in VAD device design, there were some limitations in performance: the metal cup caused scalp bruising and when posterior delivery was attempted, the device would fail due to leverage movement caused by the metal chain onto the suction tube.^71^ Stöstedt and Bird^68^ addressed these problems through the design of a shallower profile cup for easier vaginal insertion and a neoprene or polypropylene mesh inlay for less traumatic scalp interaction. Bird also emphasised the need to place the cup over the flexion point in the median position to promote flexion towards the narrowest diameter of the foetal head. To facilitate this, he separated the suction and traction ports, moving the suction port to the side of the cup, enabling placement over the flexion point even in problematic positions.^45^ Bird's modification of the Malmstroem cup, coupled with the emphasis on correct placement over the flexion point, and his advice on the finger-thumb traction technique remain the basis of best practice in vacuum-assisted delivery. Further variations on this design were introduced by O’Neil et al. in 1987 replaced the chain attachment with a curved metal rod linked to the cup by a ball joint, intending to improve manipulation.^72^ However, across three studies (627 women) results showed there was no difference in maternal and neonatal outcome between these three variations on a metal cup design.^73^

**Figure 6.**
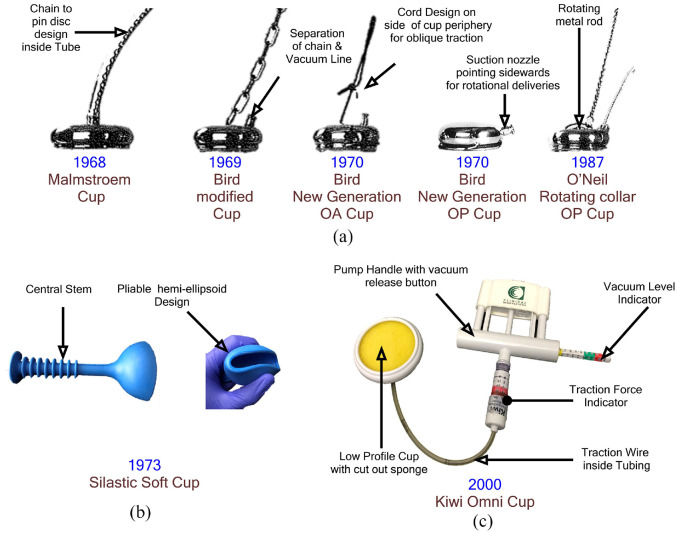
Evolutionary trail of modern VAD devices: (a) Metal cups,^71^ (b) Kobayashi silastic cup and (c) Single use instrumented devices-Kiwi Omni Cup©.

Driven by concerns that rigid metal cups could lead to scalp trauma on the infant, the 1970s saw the introduction of pliable cups made from elastomeric materials.^53^ Kobayashi introduced a VAD system consisting of a hemi-ellipsoid Silastic™ cup with a 65 mm opening and a central stem (see Figure 6). The compliance of the elastomeric material allowed it to be folded to ease insertion with minimal maternal trauma.^71^ Other VAD devices, such as the Menox Silc™ cup and the Mityvac™ cup, employed similar approaches and used elastomers to provide a ‘soft’ inner cup which helped to enhance contact area between scalp and cup. Obstetricians using these devices reported well-controlled delivery with minimal maternal trauma.^74^ However they also showed significant limitations because they could only be used to assist low to outlet delivery stations (+3 or +5, see Figure 1) and were not advised for deliveries requiring rotation of the baby’s head due to their compliant nature inhibiting the application of torque to effect head flexion.

The last major innovation to VAD systems came during the 1990s, catalysed by moves to reduce transmissible infection through single-use instrumentation. In response, two single use VADs, complete with integrated hand-pumps entered the market; the MityOne™ (also known as the Mystic II) by Cooper Surgical Ltd and the Kiwi Omni Cup by Clinical Innovations Inc. The MityOne™ has two models with different cup designs, the M-Style (mushroom-shaped cross-section) cup is a clear polyethylene cup with a flexible coupling to account for bending during delivery and the MitySoft™ which features a more rigid shaft but a larger softer cup suited for low-station delivery.^75–77^ The Kiwi Omni cup was developed at a similar time and comprises a low-profile rigid plastic cup accommodating an integrated suction tube connected to a manual hand-pump via a flexible wire. Like the ‘air tractor’, a sponge is placed inside the cup to avoid obstruction to the vacuum inlet. The handle also features indicators to display vacuum-level and traction level during use.^78,79^ However despite the addition of instrumentation, the device has not shown significant improvement on delivery rate success in comparison to older cups (e.g. Malmstroem or Bird’s cups) and actually presents higher rates of cup detachment (up to 21%).^79–81^

The evolution of VAD devices described here provides an insight into the motivations driving change and the relatively modest innovations which have occurred as a result. Key advances addressed easing cup insertion and handling inside the birth canal, reduction of device failure rate and the use of instrumentation to help regulate the procedure. However, much of this evidence is circumstantial and there is no direct literature on the assessment of VAD device design attributes of commercially available VAD devices. With the growing popularity of VAD, there is an urgent need to evaluate the performance of design attributes of VAD devices especially when little is known on how these factors contribute to maternal/foetal trauma during operational device failure.

## VAD mechanics & performance

Understanding the mechanics of VAD use is central to inform improvements in both device design and clinical utility. However, much like the limited evidence available to explain VAD device evolution, there is a paucity of literature on how these systems behave during their interaction with the scalp of a baby and how device performance could be quantified.

**Figure 7.**
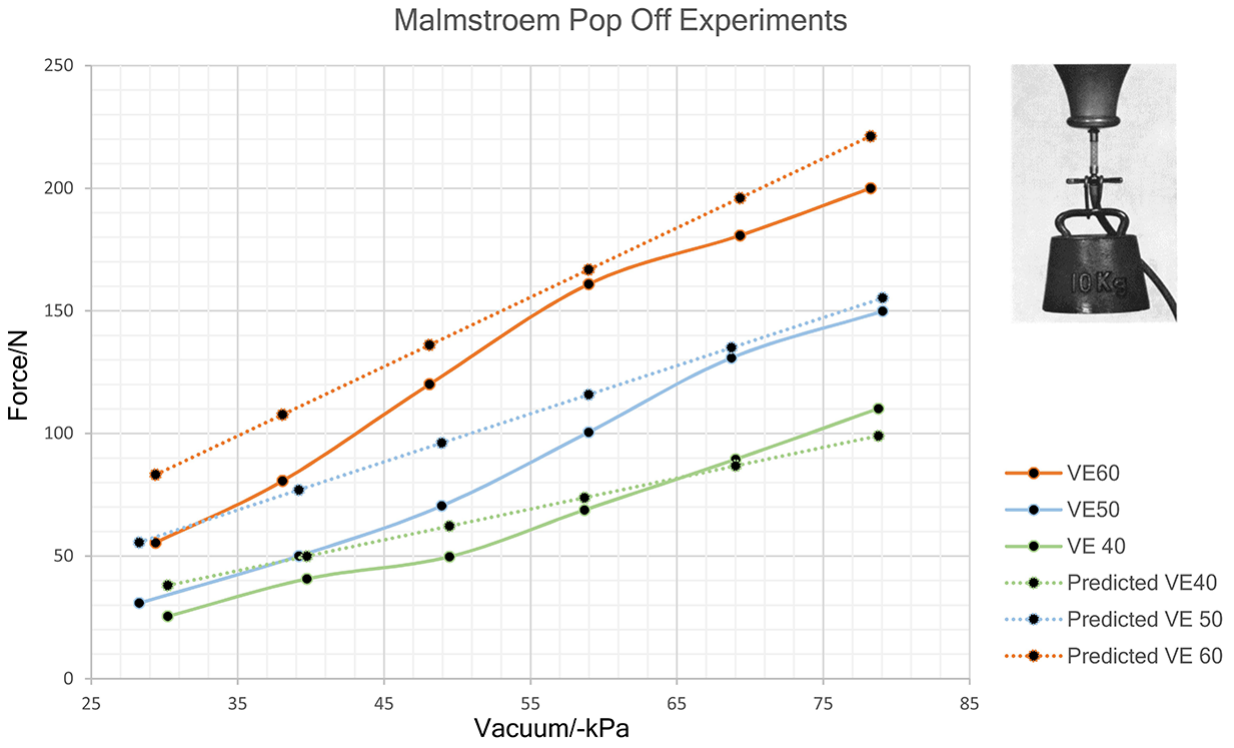
Replotted traction experiments by Malmstroem^38^ VE60:60 mm diameter cup, VE50:50 mm diameter cup, VE40: 40 mm diameter cup. Predicted curves displays force values modelled vacuum induced multiplied by the contact cross-sectional area of cup onto scalp.

The most expansive research in this area was conducted by Malmstroem to inform development of his VAD system in the 1960s. Studies focussed on optimising the maximum traction forces the VAD can exert until cup detachment (pop-off). A rubber ball was used to simulate the scalp of the baby to which a VAD was attached and loaded using fixed weights. The study investigated the effect of applying different levels of vacuum (30–80 kPa) across a range of cup diameters (40–60 mm), as shown in Figure 7.^38^ The results are intuitive, showing increased levels of maximum tractions as a function of increasing vacuum and cup diameter.

**Figure 8.**
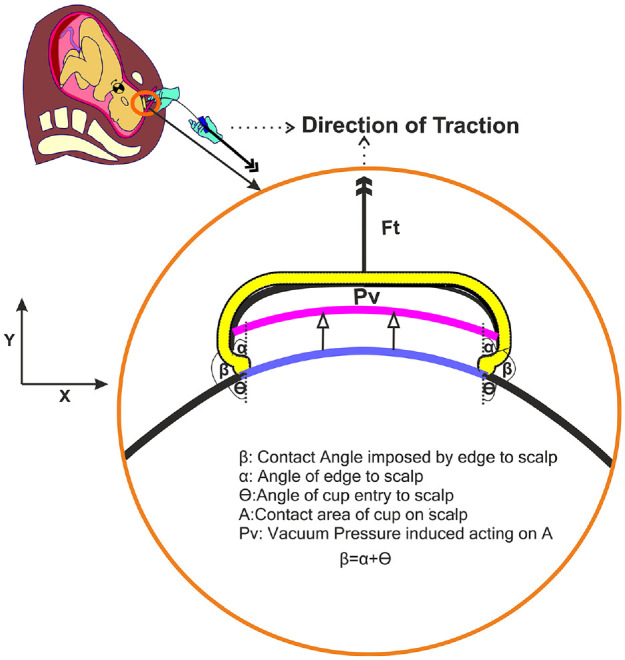
Attachment of cup onto flexion point and creation of chignon.

Based on these experiments and his personal experience, Malmstroem recommended that his VAD system would be safe and clinically effective if the vacuum is achieved at a rate of −20 kPa/min up to a maximum of −80 kPa.^82^ The rationale was that this would allow the soft tissue layers of the scalp to conform inside the hemispherical suction cup, thereby creating a chignon (Figure 8). However, Svenningsen challenged this approach, proposing that the vacuum be rapidly applied to −80 kPa as a time saving measure. This was supported by a study (*n* = 60) which showed no difference in VAD traction forces compared to a slower vacuum rate, although consideration of how this may result in tissue trauma was not detailed.^83^

Litigation related to malpractice in VAD has catalysed research into improved safety and clinical outcome, which have typically been associated with the characteristics of applied traction.^84^ Vacca reported that a traction force of 115N would be sufficient for successful delivery in 80% of cases but that the traction should not exceed 135N as this would significantly increase the risk of maternal sphincter damage and scalp injury to the baby.^41^ This is supported by an investigation by Saling into the traction forces recorded during clinical use of the Malmstroem device (60 mm Malmstroem Cup) which reported a maximum force of 125N for successful delivery. This revealed that neonatal birthweight and progression of labour has a causative link with the tractive force required. Revealingly it also highlighted a need to investigate the effect of applied traction on foetal morbidity (e.g. traumatic lesions and foetal head compressions).^85,86^ Building on this work, Muise et al. investigated the effect of applying angular traction using a range of modern VAD devices. These experiments used a scalp model (ex vivo canine hind quarters) and found that the application of angled traction resulted in a linear reduction in the safe maximal tractive force which could be applied.^87–89^

Unintentional cup detachments are the most prevalent clinical adverse event in VAD and was investigated by Bestgen et al. on porcine belly.^90^ This work introduced the concept of defining a maximum traction force to avoid both scalp trauma and cup detachment, now seen in instrumented VAD systems like the Kiwi OmniCup™.^46^ However, definition of what constitutes a safe level of traction force remains subjective and strongly dependent on device type.^91^

The studies presented here represent the most significant contributions in reporting the mechanics of VAD use. Although these are valuable, it is evident that the evidence base is poorly developed and the parameters surrounding safe operation of VAD are not well understood.

## Discussion

Since its introduction, VAD has established itself as a vital tool in the limited array of choices available to clinicians when complications occur in vaginal delivery. The underlying approach, to create a negative pressure against the baby’s exposed scalp which can support the application of assistive force, is well-suited to the clinical workflow and has remained fundamentally unchanged through the history of VAD systems. Nevertheless, VAD technology has evolved over time with key drivers being increased safety (e.g. trying the use of softer materials for the cup), ease of use (e.g. lower profile cups to facilitate placement), reduce maternal trauma and prevention of adverse events (e.g. repositioning cables for rotational delivery). Latterly there has also been the introduction of single-use systems and a focus on feedback mechanisms to inform best practice (e.g. alarms to alert the clinician to loss of suction^92–94^ and force sensors to detect the level of traction^62,95^).

It is questionable if these features and development are clinically valuable, or rather serve to provide product differentiation in a highly competitive and risk-averse commercial market. This perhaps best explains the incremental nature of innovation in VAD systems to date where it is difficult to obtain the engineering knowledge necessary to inform and justify more radical design changes and the potentially expensive regulatory approval they would incur. Nevertheless, the clinical evidence-base provides a strong argument that more significant innovation is required to make VAD systems safer and easier to use. Accordingly, these must be informed by a more rigorous evidence-base, in particular on the biomechanics of VAD systems and how these relate to clinical outcomes.

Clinical training is fundamental to ensure safe and consistent application of VAD. There have been considerable efforts to improve the proficiency of obstetric trainees through simulation-based workshops focussed on cup placement and the theory of vacuum delivery.^96^ However, there remains a lack of knowledge to inform best practice, in particular understanding what constitutes safe application of a vacuum (in terms of rate, magnitude and time) and tractive force (in terms of magnitude and rate). For instance, increasing vacuum rate may reduce the time required to perform a delivery but bring increased maternal distress and foetal trauma; mechanical interactions between these phenomena are important but less well researched than some of the clinical factors and subsequent complications. It is also notable that the use of any assisted delivery system has the potential to impact on both mother and baby. Yet very few device comparison studies have been conducted with only a select few reporting on trauma inflicted to the baby’s scalp.^97^ This provides an opportunity to create more robust training material on understanding how a VAD device interact with the foetal scalp head during an instrumental delivery allowing the clinician to better understand the performance of their device in different conditions and make them more adept in their decision-making process to perform safer VAD.

It is likely that the challenge of investigating the mechanics and use of VAD systems has been a key limiting factor in this field. Obtaining data from real cases is difficult and inherently limited in scope. Accordingly, the development of appropriate models is critical to help advance understanding. The current literature has reported the use of scalp models made from rubber and a variety of ex vivo tissues, but there is little discussion of how well these represent the anatomy of a baby’s head, the geometry and mechanical properties of the different soft tissue layers, together with the presence of interstitial fluids, all of which govern the compound behaviour of the scalp tissue during the application of vacuum and traction. Employing a standardised model and approach to evaluate devices and their failure modes could help inform clinicians on how and when to use those devices.

Development of improved VAD models will provide a foundation with which to improve our limited understanding of VAD biomechanics. In particular, the cup-scalp interface is poorly understood but fundamental to device performance (ability to apply traction) and clinical outcome (i.e. scalp trauma). The mechanics of the cup-scalp interlock require improved definition, in particular how the convex cup profile affects chignon formation, the degree to which this mechanical interlock contributes to support traction and what stress and strain regimes are imposed on the scalp tissue as a result. Understanding the dynamics of chignon formation would also allow investigation into how the vacuum should be applied (rate and magnitude) and how it should be maintained over time. Interlinked with these factors are the mechanical properties of the cup which will dictate the relative level of scalp and cup deformation (and thus stress at the interface) during use. These properties have been explored (through metal, plastic and elastomeric cup designs) but without rigorous quantification of the resultant performance. Furthermore, the mechanics at the skin-cup interface should not be neglected; the surface tribology will determine how the scalp moves relative to the cup during chignon formation and pop-off, while localised mechanical properties will dictate how the cup surface accommodates the presence of hair or caput (leading to potential pressure loss). Another area of impact is the application of tractive effort and how the characteristics of this relate to device performance. Although this is partially governed by the needs of the mother and baby, it remains uncertain how the magnitude and rate of traction relate to device performance and outcome. While it will be challenging to address these factors it will provide a rigorous basis for optimisation of device design and operating parameters and has the potential to inform new innovations to improve safety, such as instrumented VAD systems which guide the user to maximise performance, minimise potential device failures and improve maternal outcomes.

## Conclusion

The ease of use and lower maternal morbidity associated VAD devices can make them an appealing delivery option. However, to further improve these devices to improve factors ranging from clinical usability through to maternal and foetal morbidity, requires a better understanding of the mechanical interaction between the VAD and the foetal scalp.

Since mainstream adoption in 1968 design changes have been reported, motivated by usability enhancement for easier clinical use inside the birth canal, the desire to reduce device failure rates during deliveries involving rotation and gauging of vacuum/force feedback during traction. However, there exists a paucity of engineering understanding behind operational use of VAD devices. There is minimal evidence to inform VAD device design or clinical use and with the growing popularity of VAD, there is an urgent need to evaluate the performance of these medical devices.

This presents a real opportunity for driving research in achieving a better understanding of VAD operation from an engineering perspective. Supported evidence to quantify physical parameters such as safe tractive forces as well prevention of unintentional cup detachments could influence VAD best practice and perhaps provide insight on how future devices can be engineered to make VAD less traumatic.

## Appendix

**Appendix:** Glossary of Terms

Term	Explanation/Definition
Oedema	Oedema is any serous fluid collection in extra vascular tissue and can be the result of multiple causes e.g. infection, inflammation or trauma. During labour, the serosanguinous fluid accumulating in the subcutaneous tissue of the foetal scalp & the periosteal tissue of the foetal skull, is similar to oedema but termed caput succedaneum.
Chignon	Build-up of bloody fluid caused by induced pressure by the application of a VAD device to create cohesion between the cup effector and the scalp.
Caput Succedaneum	During labour, the serosanguinous fluid accumulating in the subcutaneous tissue of the foetal scalp & the periosteal tissue of the foetal skull, is similar to oedema but termed caput succedaneum.
Terms of delivery	UK Pre-Term: Delivery at a gestational age of 24-36 weeks and 6 days pregnancy (~11%). Full Term: Delivery at a gestational age of 37-40 completed weeks pregnancy (~80%). Post Term: Delivery at a gestational age of >40 weeks pregnancy (~10 %). USA Early term- (37 0/7 weeks of gestation through 38 6/7 weeks of gestation) Full term- (39 0/7 weeks of gestation through 40 6/7 weeks of gestation) Late term- (41 0/7 weeks of gestation through 41 6/7 weeks of gestation), Post term- (42 0/7 weeks of gestation and beyond) to more accurately describe deliveries occurring at or beyond 37 0/7 weeks of gestation.
Type of labour	Unaided/spontaneous vaginal delivery (i.e., no instruments required), In the UK an unaided or spontaneous normal delivery is usually managed by a trained midwife, without the involvement of a physician. In other parts of the developed world, physicians may routinely attend normal deliveries. In developing nations, if a woman is cared for in labour by a trained birth attendant, their skills will be more akin to that of a midwife. Iatrogenic: Medically caused. An induced labour is iatrogenic.
Gravida/Parity	Primigravida: A woman pregnant for the first time Multigravida: A woman pregnant multiple times
Cervical Effacement & dilation	As labour progresses the cervix shortens and thins out, a process termed effacement. The cervix also stretches open, termed dilatation, allow the passage of the baby through the birth canal.
Flexion Point	Located 3 cm forward of the posterior fontanelle along the sagittal suture and is the ideal application point for VAD to be placed to maintain flexion of the foetal head during traction
Fontanelles	Gaps between the foetal skull bones which allows the passage of the baby through the maternal pelvis
Presentation of foetal body	Cephalic: Baby’s head presenting downwards to pelvis Breech: Baby’s buttock presenting downwards to pelvis
OA, OT, OP position: Orientation of foetal head	OA: Occipito anterior- Occipital bone positioned towards mother’s belly OP:Occipito posterior- Occipital bone positioned away from mother’s belly OT: Occipito transverse- Occipital bone positioned sideways to the mother’s belly
Stations of Delivery (-1 to +5)	The station of delivery is used to describe the position of the presenting part of the baby in relation to a bony anatomical landmark in the maternal pelvis, the ischial spines. This is conventionally measured in centimetres, above (minus) or below (plus) the ischial spines. The clinical description “-3 above spines” would therefore represent a high head, the leading edge of which sitting at a plane where it is only just entering the maternal true pelvis. +1 represents a head which has advanced 1cm beyond the plane of the ischial spines.
Moulding	Suture apposed (+1): Foetal head bones touching and not overlapping Sutures overlapped but reducible (+2): Foetal head bones gently overlapping and can be restored back to position with a gentle touch Suture overlapped and not reducible (+3): The extreme case leading to overlapping of bones not easily restored to position.
Serosanguinous	Referring to blood and the serum liquid part of blood.
Introitus	Entrance to the vaginal canal
Ischial spine	Anatomical bony landmark in pelvis
Mento-vertical diameter	Engagement diameter of baby head during cephalic delivery
Subgaleal haematomas	Bleeding in between the skull periosteum and the scalp galea aponeurosis
Episiotomy	Surgical incision of the perineum and posterior vaginal wall
RCOG	Royal College of Obstetricians and Gynaecologists
ACOG	American College of Obstetricians and Gynaecologists
